# An explanation for negligible senescence in animals

**DOI:** 10.1002/ece3.8970

**Published:** 2022-06-06

**Authors:** Canwei Xia, Anders Pape Møller

**Affiliations:** ^1^ 47836 Ministry of Education Key Laboratory for Biodiversity and Ecological Engineering College of Life Sciences Beijing Normal University Beijing China; ^2^ Ecologie Systématique Evolution Université Paris‐Sud, CNRS AgroParisTech, Université Paris‐Saclay Orsay Cedex France

**Keywords:** gompertz model, recovery probability, reported lifespan, senescence, weibull model, wild animals

## Abstract

Negligible or negative senescence occurs when mortality risk is stable or decreases with age, and has been observed in some wild animals. Age‐independent mortality in animals may lead to an abnormally long maximum individual lifespans and be incompatible with evolutionary theories of senescence. The reason why there is no evidence of senescence in these animals has not been fully understood. Recovery rates are usually very low for wild animals with high dispersal ability and/or small body size (e.g., bats, rodents, and most birds). The only information concerning senescence for most of these species is the reported lifespan when individuals are last seen or caught. We deduced the probability density function of the reported lifespan based on the assumption that the real lifespan corresponding to Weibull or Gompertz distribution. We show that the magnitude of the increase in mortality risk is largely underestimated based on the reported lifespans with low recovery probability. The risk of mortality can aberrantly appear to have a negative correlation with age when it actually increases with increasing lifespan. We demonstrated that the underestimated aging rate for wild animals with low recovery probability can be generalizable to any aging models. Our work provides an explanation for the appearance of negligible senescence in many wild animals. Humans attempt to obtain insights from other creatures to better understand our own biology and its gain insight into how to enhance and extended human health. Our advice is to take a second glance before admiring the negligible senescence in other animals. This ability to escape from senescence is possibly only as beautiful illusion in animals.

## Graphical abstract

We provided a reasonable explanation for the negligible senescence in wild animals. By employing mathematical models and simulated data, we showed that the magnitude of increase in mortality is largely underestimated based on the reported lifespan. Mortality rate can show a descending trend toward old age even if it subsequently increases with age.

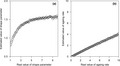

## 1 INTRODUCTION

Senescence, the process of the decline in physiological traits, vitality, and reaction capability, can be reflected in the form of an increase in mortality risk positively correlated with age (Finch, 1990). However, there is a long history of the lack of senescence in wild animals. Pearl (1928) and Deevey (1947) thought most animals in nature have straight or concave survivorship curves, “to be alike in suffering a constant risk to death from early adult life to the end of the life span.” Lack (1943a, 1973b) and Pinder et al. (1978) noted that mortality is nearly stable or decreasing when approaching old age in 8 wild bird species: blackbird *Turdus merula*, song thrush *Turdus ericetorum*, starling *Sturnus vulgaris*, lapwing *Vanellus vanellus*, woodcock *Scolopax rusticola*, black‐headed gull *Larus ridibundus*, American robin *Turdus migratorius*, and herring gull *Larus argentatus*. Even in recent studies with high‐quality longitudinal field records, there is also apparent evidence for negligible or negative senescence in mammals and birds, for example, grassland rodents *Peromyscus* spp. (Slade, 1995), common pochards *Aythya ferina* (Nichols et al., 1997), barn owls *Tyto alba* (Altwegg et al., 2007), sparrowhawks *Accipiter nisus*, and collared flycatchers *Ficedula albicollis* (Jones et al., 2014).

Negligible or negative senescence in wild animals has been questioned (Gaillard & Lemaître, 2020; Nussey et al., 2013), as it can lead to unreasonable predictions (e.g., inconceivable maximum lifespans) and be incompatible with evolutionary theories of aging. Taking into account the assumption of constant mortality, Botkin and Miller (1974) predicted the potential longevity of several bird species. For example, in a population with 1000 individuals, the predicted maximum longevity is 170 years for herring gulls, 102 years for both gannets *Morus bassanus* and fulmars *Fulmarus glacialis* (Botkin & Miller, 1974). However, according to the ringing recoveries from EURING (https://euring.org/data‐and‐codes/euring‐databank), the observed maximum longevity for herring gulls, gannets, and fulmars is 34, 37, and 43 years, respectively. Clearly, there are huge gaps between the predicted and observed maximum longevities in these species. From an evolutionary perspective, mutations that are detrimental late in life will tend to equilibrate at higher frequency than in early life, causing the force of natural selection to weaken with age (Medawar, 1952). Increasing mortality can also result from the pleiotropic gene effects, positive genetic effects in early life while negative effects in late life (Williams, 1957). Both of these evolutionary theories predict that senescence is an inevitable process for organisms (Hamilton, 1966).

In accordance with the predictions of evolutionary theories, there is an abundance of evidence that shows an increase of mortality risk toward old ages in both wild animals, especially mammals and birds (Lemaître et al., 2020; Nussey et al., 2013), and zoo animals (Peron et al., 2019; Tidière et al., 2016). However, the evidence for negligible or negative senescence in some wild animals has yet to be fully addressed or explained (Baudisch, 2011; Bernard et al., 2020; Jones et al., 2014). In this study, we employed mathematical models and simulated data to show that the magnitude of increase in mortality is largely underestimated based on reported lifespans with low recovery probability. Mortality risk can show a descending trend toward old age even if it subsequently increases as an organism ages. Our results reveal that the evidence of negligible or negative senescence is probably an illusion in animals.

## 2 MATERIALS AND METHODS

Senescence depends on both pace (i.e., the time scale on which mortality progresses) and shape (i.e., how sharply mortality changes with age) (Baudisch, 2011). Within a given population, the shape of senescence can be directly displayed by the plot of mortality risk against age or reflected by the parameters (or the combinations of parameters) in aging models (Ricklefs & Scheuerlein, 2002; Ronget et al., 2020). In this study, we focused on the shape of senescence—mortality changes with age—based on two widely employed aging models: the Weibull and Gompertz models. Both these models target the changes of mortality with age from an age of onset of senescence, commonly assumed to correspond to the age at first reproduction (Pinder et al., 1978; Wrycza et al., 2015). We extend the main results to any other aging models in the discussion.

### 2.1 The frame of Weibull and Gompertz model

The Weibull model provides a close approximation to the distribution of the lifetime for the object consisting of many parts, which experiences death when any of its parts fails (Rinne, 2009; Sharif & Islam, 1980). This model is widely used to assess biological phenomena, especially in birds (Pinder et al., 1978; Ricklefs & Scheuerlein, 2002). In the Weibull model, the change in mortality risk (*m*) as a function of age (*x*) can be modeled by the expression:

(1) $$m\left(x\right)=m_{0}+\frac{c}{b}*{\left(\frac{x}{b}\right)}^{c‐1}$$

In Equation 1, *m*_0_ is the age‐independent mortality risk; *c* is the shape parameter reflecting the age‐dependent change in mortality risk; *b* is the scale parameter linked with the pace of aging; and *x* is the age after the onset of the senescent stage. The corresponding probability density function of lifespan is

(2) $$\begin{array}{c} f\left(x\right)=m\left(x\right)*\exp\left[‐\int_{0}^{x}m\left(x\right)\cdot dx\right]=\left[m_{0}+\frac{c}{b}*{\left(\frac{x}{b}\right)}^{c‐1}\right]\\ \;*\exp\left[‐m_{0}*x‐{\left(\frac{x}{b}\right)}^{c}\right], \end{array}$$

and the survivorship at age (*x*) is

(3) $$s\left(x\right)=\exp\left[‐\int_{0}^{x}m\left(x\right)\cdot dx\right]=\exp\left[‐m_{0}*x‐{\left(\frac{x}{b}\right)}^{c}\right].$$

The Gompertz model assumes an exponential increase in mortality with age. This model is the most popular and satisfactorily describes the shape of age‐specific mortality changes in humans (Gavrilov & Gavrilova, 2015) and other mammals (Ronget et al., 2020). For comparing with the previous research (Ronget et al., 2020; Tidière et al., 2016), Gompertz–Makeham model, which include a constant reflecting the age‐independent mortality risk, was used. In the model, the change in mortality risk (*m*) as a function of age (*x*) can be modeled by the expression

(4) $$m\left(x\right)=m_{0}+\theta *\exp\left(\lambda *x\right)$$

In Equation 4, *m*_0_, named as Makeham term/constant, reflects the age‐independent mortality risk; *θ* is the initial mortality at the onset of senescence; *λ* reflects the rate of aging (Baudisch, 2011; Finch, 1990); and *x* is the age after the onset of the senescent stage. The corresponding probability density function of lifespan is

(5) $$\begin{array}{c} f\left(x\right)=\left[m_{0}+\theta *\exp\left(\lambda *x\right)\right]\\ \;*\exp\left\{‐m_{0}*x‐\frac{\theta }{\lambda }*\left[\exp\left(\lambda *x\right)‐1\right]\right\}, \end{array}$$

and the survivorship at age (*x*) is

(6) $$s\left(x\right)=\exp\left\{‐m_{0}*x‐\frac{\theta }{\lambda }*\left[\exp\left(\lambda *x\right)‐1\right]\right\}.$$

**FIGURE 1:**
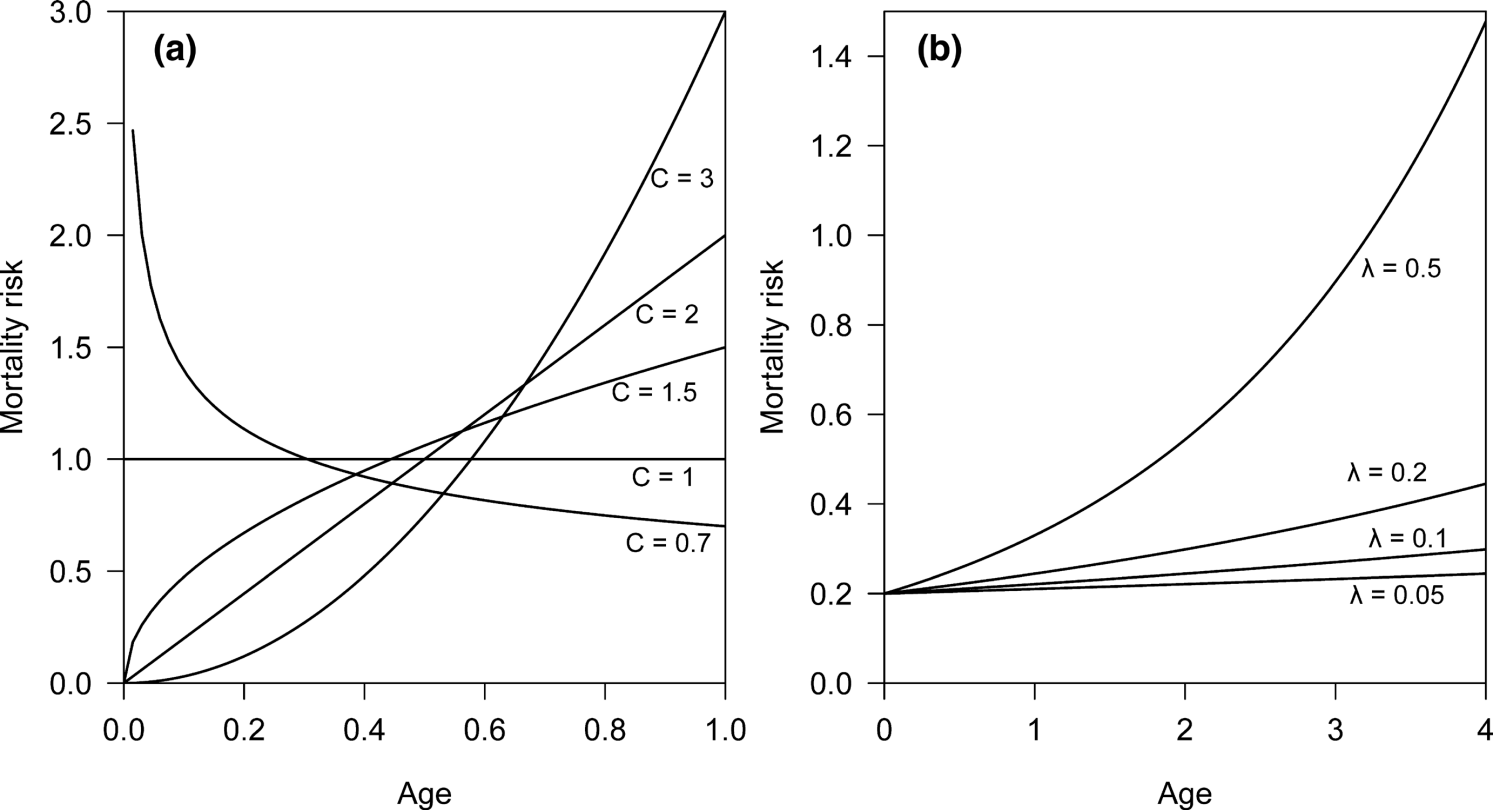
(a) The change in mortality risk against age relates to parameter *c* in the Weibull model, with a decreasing mortality risk (*c* < 1), constant mortality risk (*c* = 1), decelerated increase in mortality risk (1 < *c* < 2), linear increase in mortality risk (*c* = 2), and accelerated increase in mortality risk (*c* > 2). (b) The change in mortality risk against age relates to the parameter *λ* in the Gompertz model, with the more accelerated increase in mortality risk for a larger *λ* value

For simplicity, we first omit the term *m*_0_, which determines mortality in the early subadult stage and has little influence on the mortality toward old age, and then consider its (*m*_0_) effect on the changing of mortality risk. Following Rinne (2009) and Marshall and Olkin (2007), we used the symbols (*b*, *c*) for parameters in the Weibull model and symbols (*θ*, *λ*) for parameters in the Gompertz model. In practice, parameter *c* in the Weibull model and parameter *λ* in the Gompertz model reflect the change in the age‐specific mortality risk (Figure 1). In theory, the parameters *c* and *λ* can be estimated by observing lifespan from a sample of individuals.

### 2.2 Real lifespan and reported lifespan

It is difficult, if not impossible, to know the real lifespan of many wild animals. In field studies, lifespan data is mostly collected using the mark‐recapture method (Pradel, 1996). The longevity of individuals is recorded when they are last seen or caught, with the unknown remaining lifespan after that. The longevity collected in field studies is termed “reported lifespan” to distinguish it from the organism's actual lifespan (Moorad et al., 2012; Xia & Møller, 2021). For wild animals with high dispersal ability (e.g., birds, bats), the recovery rates are usually very low; for example, <1% of banded birds tend to be recovered (Cleminson & Nebel, 2012). When the recovery probability is <10%, which is the typical case for most bird species according to the ringing recoveries from EURING (https://euring.org/data‐and‐codes/euring‐databank), the probability of an individual being recaptured twice is <1%. Thus, we can safely assume that a marked individual can only be found at most once after marking. We also assume that the time when a particular individual is recovered belongs to a uniform distribution, and the domain is its real lifespan.

Under the above prerequisites, given that the real lifespan is *x*, the probability density function of the reported lifespan (*y*) is

(7) $$f_{Y|X}\left(y|x\right)=\frac{1}{x},\;\text{for}\;y\leq x.$$

Equation 7 means that an individual who died at age (*x*) could be observed at any time before age (*x*) but could not be observed after age (*x*). Using the condition probability formula, we can obtain the joint probability density function for the real lifespan (*x*) and reported lifespan (*y*)

(8) $$f_{X,Y}\left(x,y\right)=f_{Y|X}\left(y|x\right)*f\left(x\right)=\frac{1}{x}*f\left(x\right).$$

Then, we can deduce the probability density function for the reported lifespan (*y*)

(9) $$f\left(y\right)=\int_{y}^{\infty }f_{X,Y}\left(x,y\right)\cdot dx=\int_{y}^{\infty }\frac{1}{x}*f\left(x\right)\cdot dx.$$

Equation 9 implies that the probability of a reported lifespan at age (*y*) is contributed by the individuals with a real lifespan (*x*) equal to or larger than age *y*. Combining Equations 2 and 9, the probability density function for the reported lifespan from the Weibull model is

(10) $$f\left(y\right)=\int_{y}^{\infty }\frac{1}{x}*\left[m_{0}+\frac{c}{b}*{\left(\frac{x}{b}\right)}^{c‐1}\right]*\exp\left[‐m_{0}*x‐{\left(\frac{x}{b}\right)}^{c}\right]\cdot dx.$$

Combining Equations 5 and 9, the probability density function for the reported lifespan from the Gompertz model is

(11) $$\begin{array}{c} f\left(y\right)=\int_{y}^{\infty }\frac{1}{x}*\left[m_{0}+\theta *\exp\left(\lambda *x\right)\right]\\ \;*\exp\left\{‐m_{0}*x‐\frac{\theta }{\lambda }*\left[\exp\left(\lambda *x\right)‐1\right]\right\}\cdot dx \end{array}$$

The corresponding survivorship and mortality risk against age for reported lifespan (*y*) can be deduced from the probability density function

(12) $$s\left(y\right)=\int_{y}^{\infty }f\left(y\right)\cdot dy$$

(13) $$m\left(y\right)=\frac{f\left(y\right)}{s\left(y\right)}.$$

### 2.3 Survivorship and mortality risk

Based on the above equations, we compare the trajectories of survivorship and mortality risk between real lifespan and reported lifespan. In both the Weibull and Gompertz models, *m*_0_, the age‐independent mortality risk only influences the intercept rather than the change in mortality risk throughout the lifespan (Equations 1 and 4). For simplicity, we first set *m*_0_ equal to 0 and then considered the non‐negative value (0.2, 0.5) of *m*_0_ and analyzed its effect on the mortality risk based on the reported lifespan. In the Weibull model, the parameter *b* is changed by using different time units (e.g., month, half year, year) and does not influence the shape of mortality risk against age. Therefore, we set *b* to 1 for simplicity. We set four values for shape parameter *c* (1.25, 1.5, 2, 3) in the Weibull model. The first two values (1.25, 1.5) correspond to a decelerated increase in mortality risk, and the last two values (2, 3) correspond to a linear or accelerated increase in mortality risk (Figure 1a). In the Gompertz model, we fixed the initial mortality at the onset of senescence (*θ*) as 0.2 and set four values for the aging rate *λ* (0.05, 0.1, 0.2, 0.5), which covers the range of the real aging rate for most mammals (Lemaître et al., 2020). As the aging rate *λ* increases, there is a more accelerated increase in mortality risk (Figure 1b).

### 2.4 Parameter estimation

The above procedures displayed survivorship and mortality risk under 4 special shape parameters *c* (1.25, 1.5, 2, 3) in the Weibull model and 4 special aging rates *λ* (0.05, 0.1, 0.2, 0.5) in the Gompertz model. To assess the properties under additional values of shape parameter *c* or aging rate *λ*, we generated series sets of reported lifespans using different values for the shape parameter *c*, ranging from 1 to 10 with increments of 0.1, and for the aging rate *λ*, ranging from 0.1 to 10 with increments of 0.1. For each set (corresponding to the unique value of shape parameter *c* or aging rate *λ*), we generated the real lifespan of 10,000 individuals from the Weibull distribution (Equation 2) or Gompertz model (Equation 5) and then obtained the reported lifespan for each individual from a uniform distribution with the domain ranging from 0 to the real lifespan of this individual (Equation 7). Based on the reported lifespan, we used maximum likelihood methods to estimate the $\hat{c}$ or aging rate $\hat{\lambda }$ value. All simulated data were generated in R software (R Core Team, 2021), with maximum likelihood methods conducted in the “EnvStats” package (Millard, 2013) and “VGAM” package (Thomas et al., 2015). We realize that it is improper to estimate the shape parameter $\hat{c}$ or aging rate $\hat{\lambda }$ from the reported lifespan, as the real lifespan, rather than the reported lifespan, belongs to the Weibull distribution or Gompertz distribution. Our aim is to demonstrate the impropriety of this estimation: the increase in mortality risk against age is largely underestimated based on reported lifespan. An alternative approach is to fit equation (e.g., linear regression) for mortality risk against age. We evaluate the inherent problems of this approach in the discussion.

## 3 RESULTS

### 3.1 Results of the Weibull model

**FIGURE 2:**
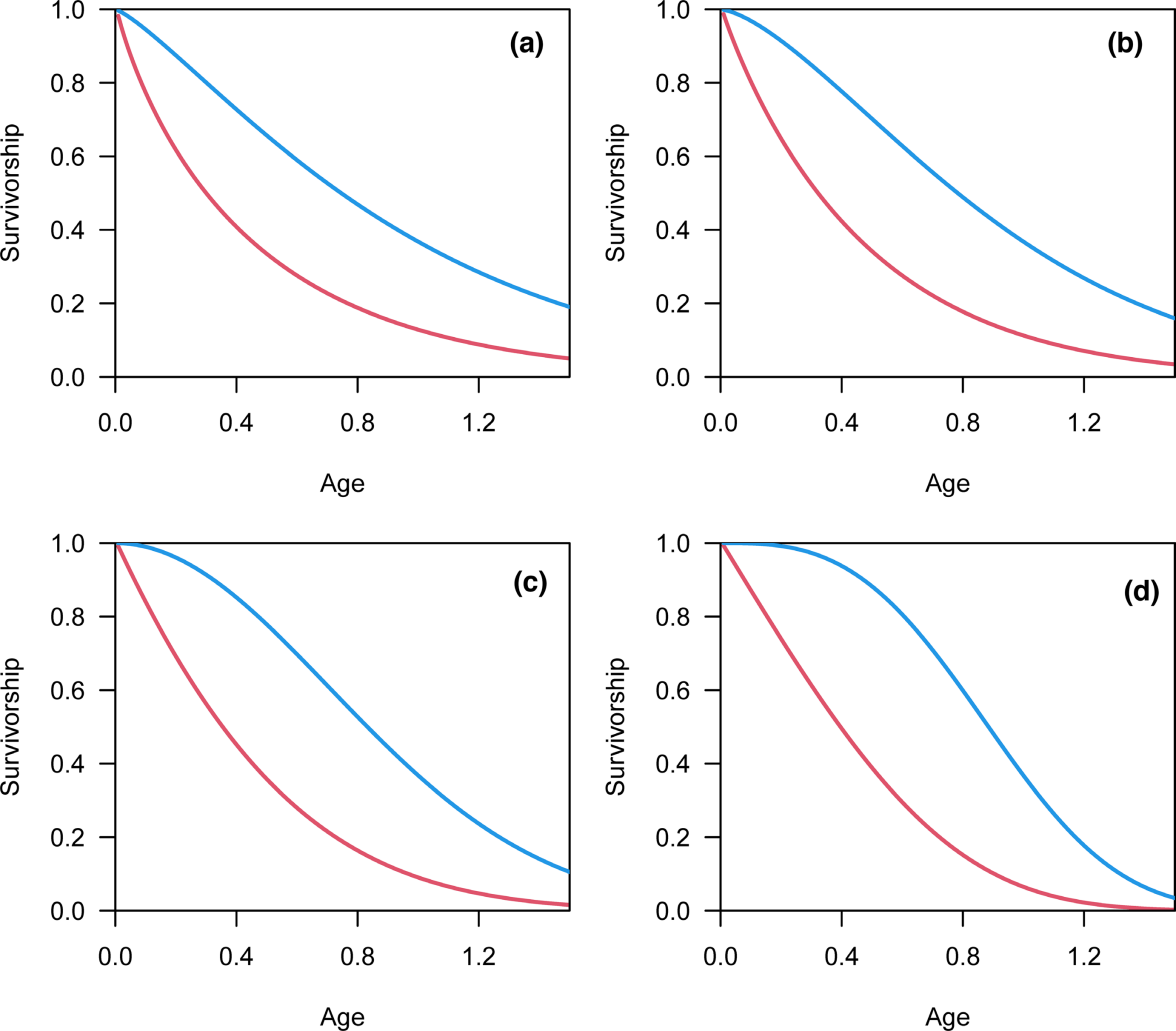
The survivorship curves based on real lifespan (blue lines) and reported lifespan (red lines). The simulated data were generated by Weibull distributions with shape parameters equal to 1.25 (a), 1.5 (b), 2 (c), and 3 (d). As the shape parameter increases, the convexity of the survivorship curve based on real lifespan (blue lines) is increasingly evident, while survivorship curves are almost straight, with a somewhat concavity, based on reported lifespans (red lines)

**FIGURE 3:**
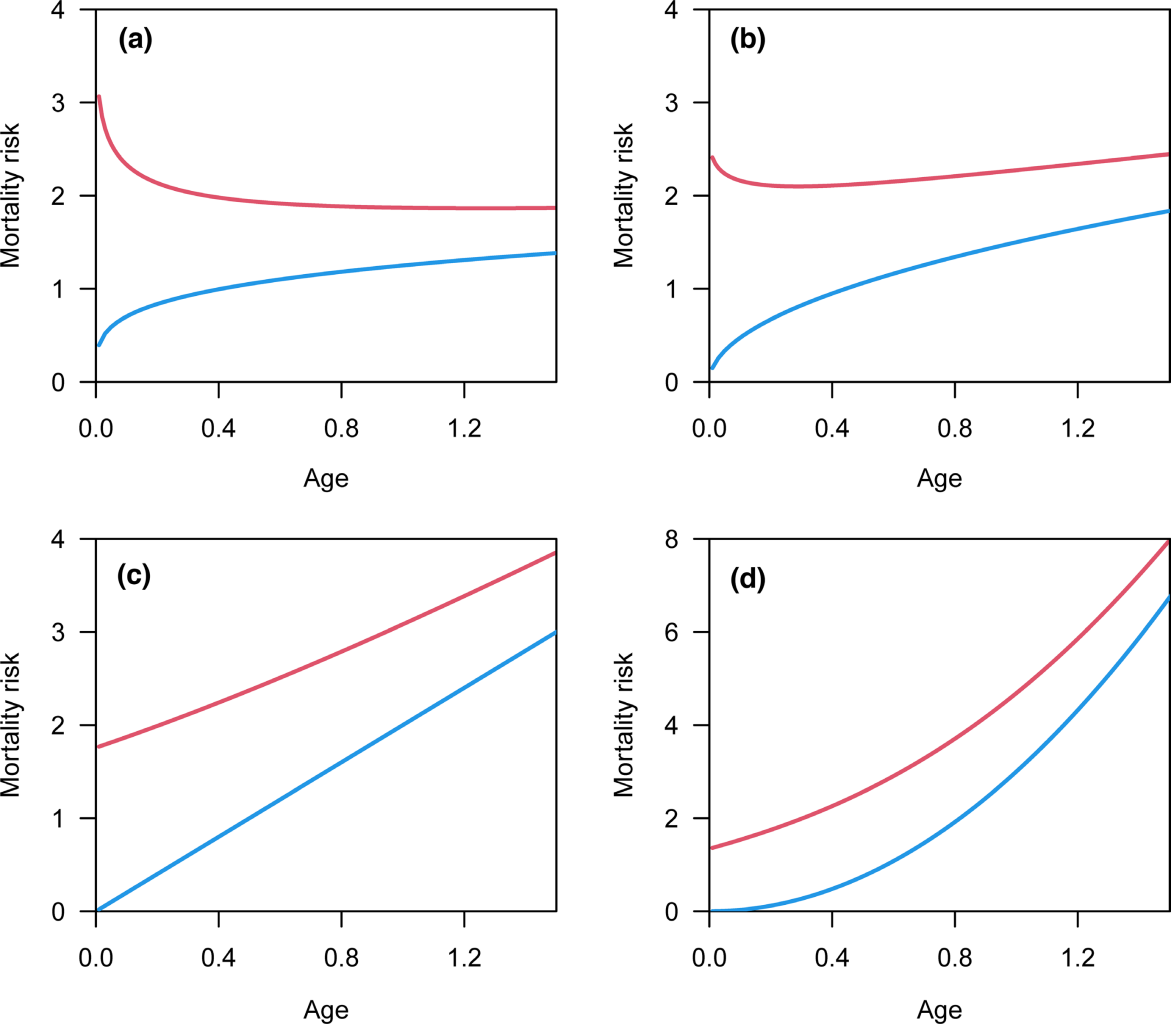
Mortality risk plotted against age based on real lifespan (blue lines) and reported lifespan (red lines). The simulated data were generated by Weibull distributions with shape parameters equal to 1.25 (a), 1.5 (b), 2 (c), and 3 (d). For the real lifespan (blue lines), the mortality risk increases with age. For the reported lifespan (red lines), the mortality risk decreases with age (a) or remains nearly constant (b). The increasing mortality risk is shown in (c) and (d); however, the magnitude of increase is less for the reported lifespan (red lines) than for the real lifespan (red lines)

As the shape parameter (*c*) used to generate simulated data increases in the Weibull model, the convexity of the survivorship curve based on real lifespan is increasingly evident (blue lines in Figure 2a–d), and the corresponding mortality risk increases with age (blue lines in Figure 3a–d). However, survivorship curves are nearly straight, with a slight concavity, based on the reported lifespan (red lines in Figure 2a–d). For the reported lifespan, the mortality risk decreases with age or remains nearly constant for relatively small *c* values (red lines in Figure 3a,b). The increasing mortality risk is demonstrated when the *c* value is relatively large; however, the magnitude of increase is still underestimated as the mortality risk at the beginning is overestimated (red lines in Figure 3c,d).

### 3.2 Results of the Gompertz model

**FIGURE 4:**
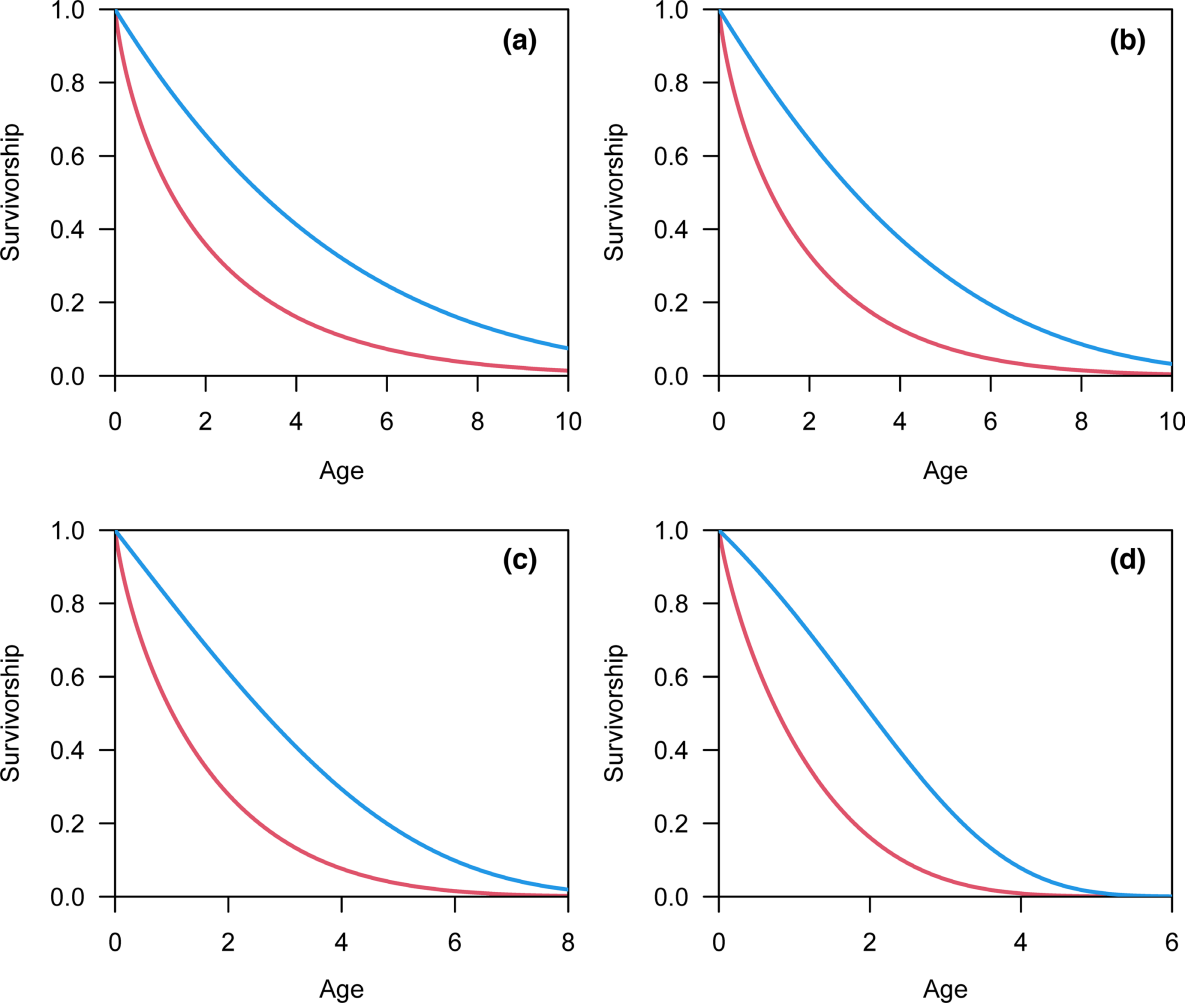
The survivorship curves based on real lifespan (blue lines) and reported lifespan (red lines). The simulated data were generated by Gompertz distributions with aging rates equal to 0.05 (a), 0.1 (b), 0.2 (c), and 0.5 (d). As the aging rate increases, the convexity of the survivorship curve based on real lifespan (blue lines) is increasingly evident, while survivorship curves are almost concave based on reported lifespans (red lines)

**FIGURE 5:**
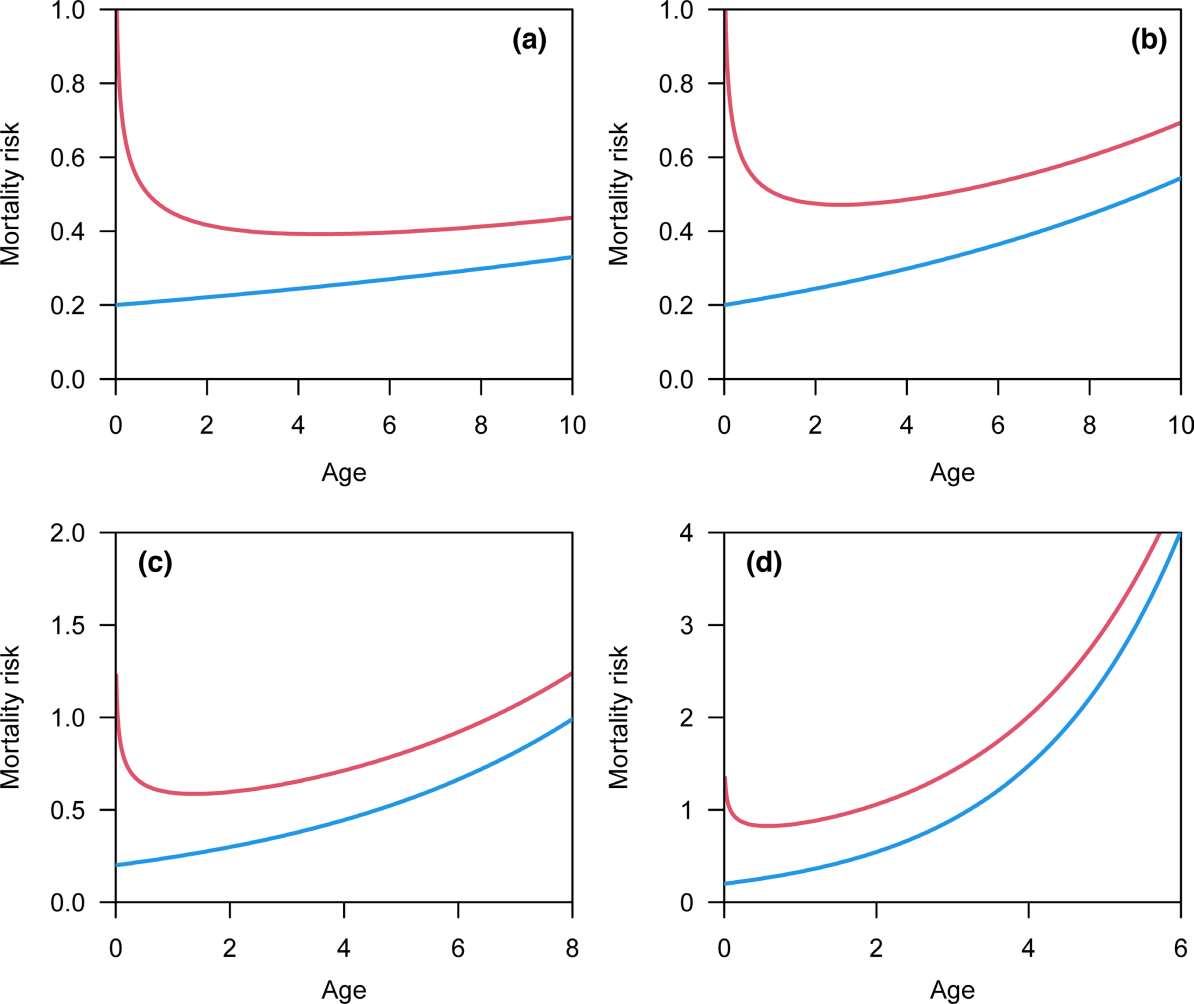
Mortality risk plotted against age based on real lifespan (blue lines) and reported lifespan (red lines). The simulated data were generated by Gompertz distributions with aging rates equal to 0.05 (a), 0.1 (b), 0.2 (c), and 0.5 (d). For the real lifespan (blue lines), the mortality risk increases with age (a–d). For the reported lifespan (red lines), the mortality risk remains nearly constant (a). The increasing mortality risk is shown in (b–d); however, the magnitude of increase is less for the reported lifespan (red lines) than for the real lifespan (red lines)

As the aging rate (*λ*) used to generate simulated data increases in the Gompertz model, the convexity of the survivorship curve based on real lifespan is increasingly evident (blue lines in Figure 4a–d), and the corresponding mortality risk increases with age (blue lines in Figure 5a–d). However, survivorship curves are concave based on the reported lifespan (red lines in Figure 4a–d). Additionally, the mortality risk remains nearly constant for relatively small *λ* values of the reported lifespan (red lines in Figure 5a). The increasing mortality risk is demonstrated when the *λ* value is relatively large; however, the magnitude of increase is still underestimated as the mortality risk at the beginning is overestimated (red lines in Figure 5b–d).

### 3.3 The influence of non‐negative *m*_0_ value

**FIGURE 6:**
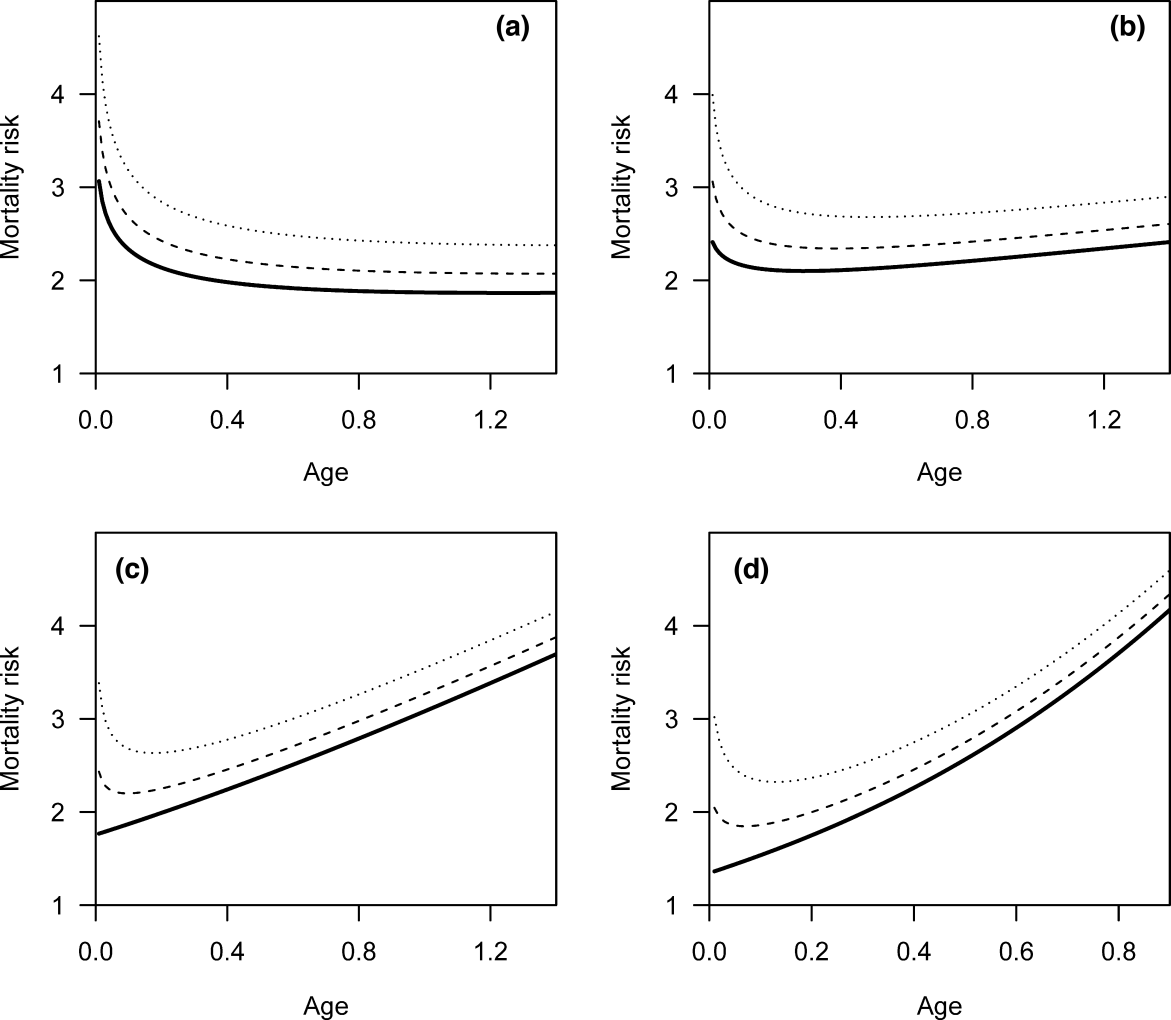
The change in mortality risk against age based on reported lifespan is not affected by *m*_0_ (i.e., age‐independent mortality risk) in the Weibull model. *m*_0_ is equal to 0 for the blank lines; *m*_0_ is equal to 0.2 for the dashed lines; *m*_0_ is equal to 0.5 for the dotted lines. The trajectories correspond to the Weibull model with shape parameters equal to 1.25 (a), 1.5 (b), 2 (c), and 3 (d)

**FIGURE 7:**
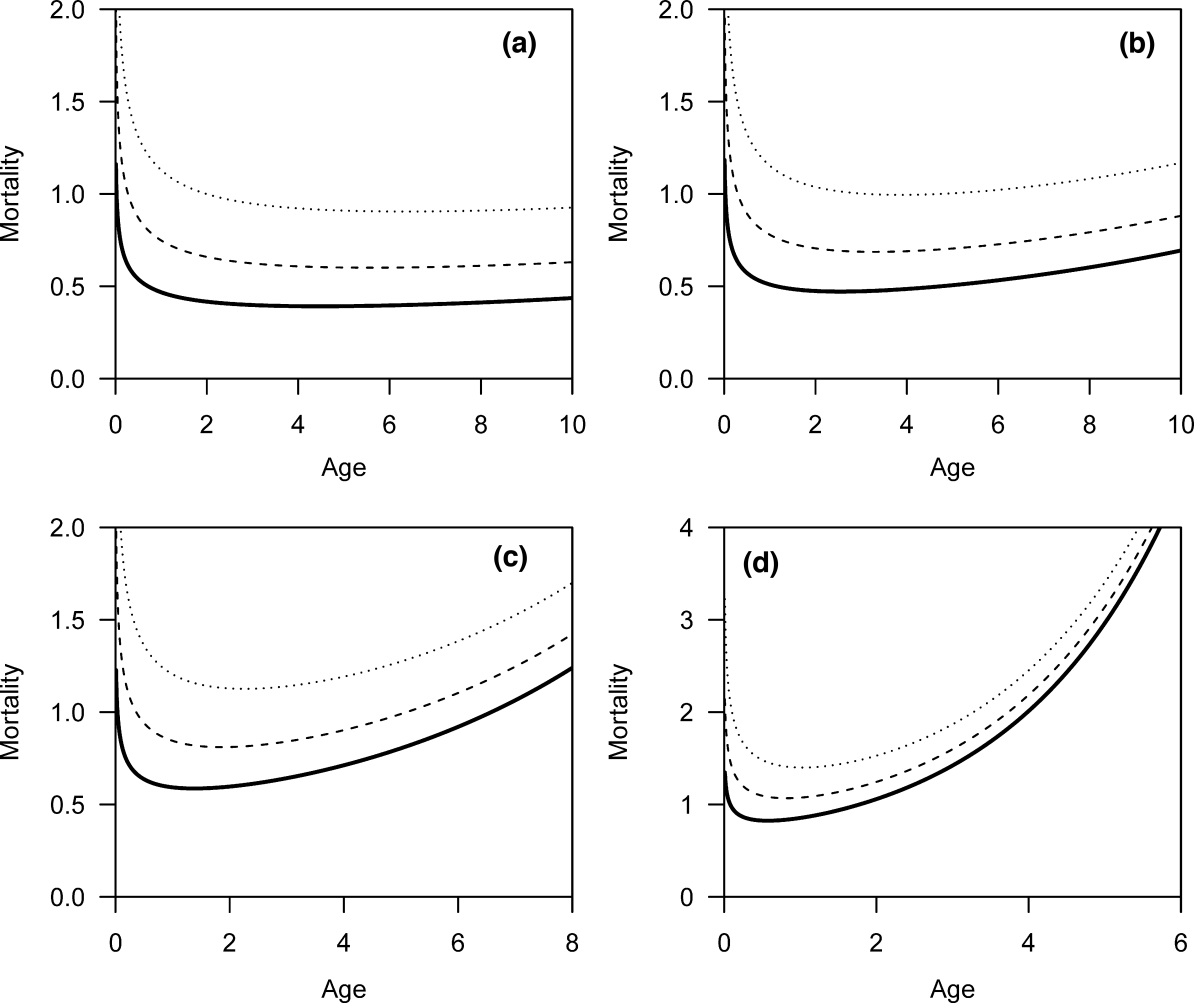
The change in mortality risk against age based on reported lifespan is not affected by *m*_0_ (i.e., age‐independent mortality risk) in the Gompertz model. *m*_0_ is equal to 0 for the blank lines; *m*_0_ is equal to 0.2 for the dashed lines; *m*_0_ is equal to 0.5 for the dotted lines. The trajectories correspond to the Gompertz model with aging rates equal to 0.05 (a), 0.1 (b), 0.2 (c), and 0.5 (d)

Theoretically, *m*_0_ could be any non‐negative value. From Equations 1 and 4, it is evident that the value of *m*_0_ has no effect on the change in mortality risk based on the real lifespan, as *m*_0_ only influences the intercept of the trajectory. This property still holds up for reported lifespan. From the trajectory of the mortality risk (Figures 6 and 7), we can see that the mortality risk at the beginning is further overestimated as *m*_0_ increases, and the trajectories are nearly parallel toward old age. Thus, the increase in mortality risk is still underestimated for nonzero values of *m*_0_ in both the Weibull and Gompertz models.

### 3.4 Parameter estimation in the model

**FIGURE 8:**
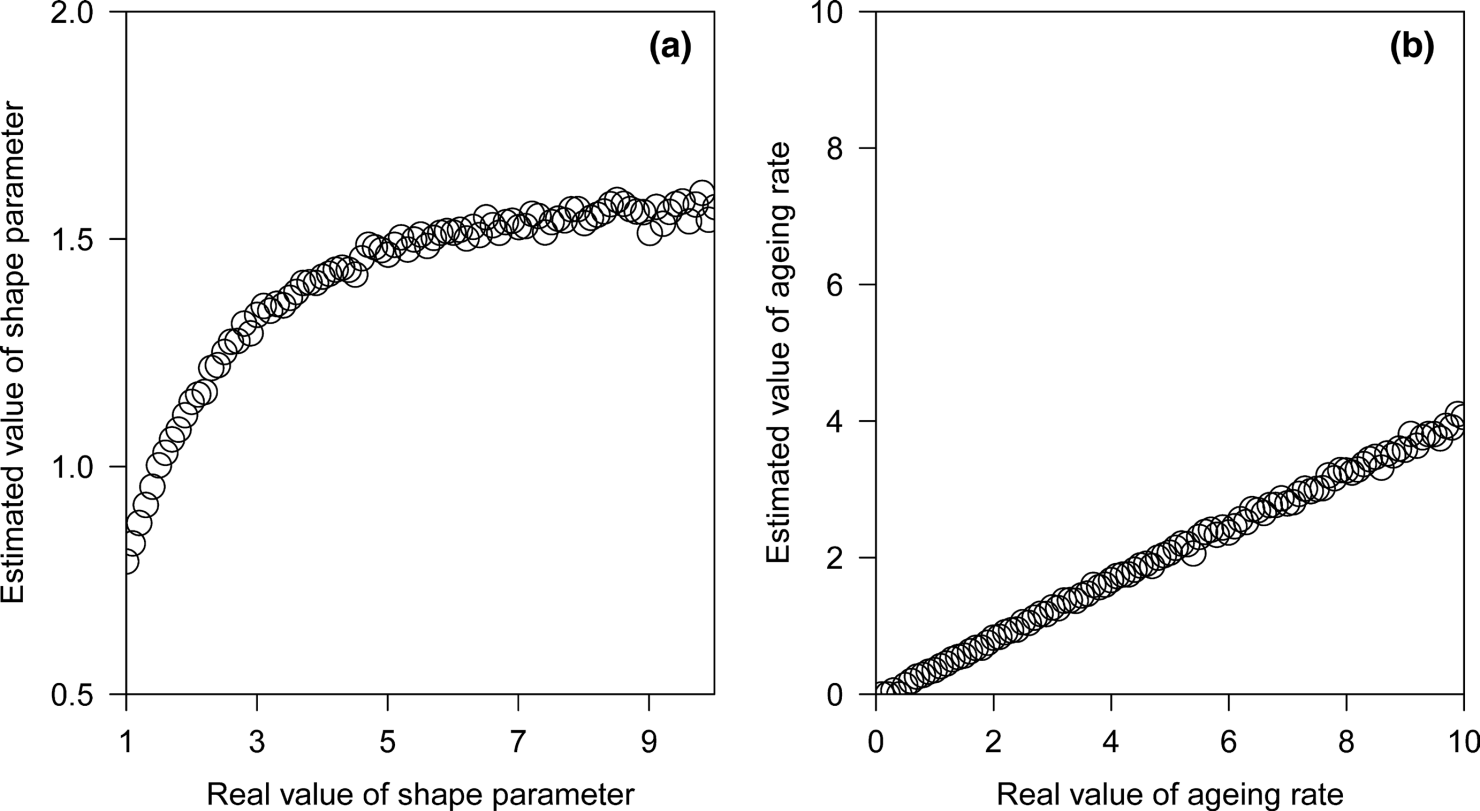
(a) In the Weibull model, the estimated value of the shape parameter *c* is underestimated based on the reported lifespan, with an upper bound of approximately 1.5. (b) In the Gompertz model, the estimated $\hat{\lambda }$ value has a linear relationship with the real *λ* value. However, the slope is <1, which indicates the underestimated value of the aging rate based on the reported lifespan

In the Weibull model, the change in mortality risk against age is reflected by the value of the shape parameter *c*. The estimated $\hat{c}$ value based on the reported lifespan is largely underestimated, with an upper bound of approximately 1.5 (Figure 8a). In the Gompertz model, there is a linear relationship between the estimated $\hat{\lambda }$ value and the real *λ* value (regression coefficient = 0.41, *t*_98_ = 201, *p* < .001). However, the slope in this linear relationship was <1 (*t*_98_ = 292, *p* < .001), which indicates the underestimated value of the aging rate based on the reported lifespan. This shows that the increase in mortality risk against age is largely underestimated based on the reported lifespan in both the Weibull and Gompertz models.

## 4 DISCUSSION

Negligible or negative senescence—a pattern of stable or decreasing mortality with age—has been apparently observed in some wild animals (Altwegg et al., 2007; Jones et al., 2014; Lack, 1943a, 1943b; Nichols et al., 1997; Pinder et al., 1978; Slade, 1995). However, age‐independent mortality may lead to an unrealistic maximum lifespan (Botkin & Miller, 1974) and run counter to evolutionary theory (Hamilton, 1966; Medawar, 1952; Williams, 1957). The inability to obtain evidence of senescence in the past was generally attributed to insufficient sample sizes in field studies (Gaillard & Lemaître, 2020; Nussey et al., 2013). However, even in pioneering research that appears to show negligible senescence in wild animals, the sample sizes can range from hundreds to thousands (Lack, 1943a, 1943b; Pinder et al., 1978), which is equivalent to the sample sizes based on captive animal research that clearly show evidence of senescence (Peron et al., 2019; Ronget et al., 2020; Tidière et al., 2016). Here, we provided an explanation for the negligible senescence in wild animals by showing that an increase in mortality risk was largely underestimated based on reported lifespans with low recovery probability.

### 4.1 Technical issues

Based on the framework of the Weibull and Gompertz models, it can be observed that the mortality risk at the start age of the senescent stage is overestimated (Figures 3 and 5), which leads to the underestimation of increased mortality risk with age. Here, we demonstrated that this property can be extended to any other aging models. Let *f_X_* (1) correspond to the probability of real lifespan in the first‐time interval. As all individuals survive at the beginning (i.e., $s_{X}\left(1\right)=1$), mortality risk in the first‐time interval based on the real lifespan is

(14) $$m_{X}\left(1\right)=\frac{f_{X}\left(1\right)}{s_{X}\left(1\right)}=f_{X}\left(1\right).$$

The mortality risk in the first‐time interval based on the reported lifespan is:

(15) $$\begin{array}{c} m_{Y}\left(1\right)=\frac{f_{Y}\left(1\right)}{s_{Y}\left(1\right)}=f_{Y}\left(1\right)=\sum_{i\;=\;1}^{\infty }\frac{f_{X}\left(i\right)}{i}=f_{X}\left(1\right)+\sum_{i\;=\;2}^{\infty }\frac{f_{X}\left(i\right)}{i}>m_{X}\left(1\right) \end{array}$$

Therefore, the mortality risk at the start age of the senescent stage must be overestimated from the reported lifespan, which can be generalizable to any aging models.

In this study, we used maximum likelihood methods to estimate the $\hat{c}$ value in the Weibull model and the $\hat{\lambda }$ value in the Gompertz model based on the reported lifespan and showed that the increase in mortality risk against age was largely underestimated (Figure 8). An alternative approach is to fit the equation (e.g., linear regression) for mortality risk against age. Here, we discussed the limitations of this approach. From Equation 9, we can get:

(16) $$\begin{array}{c} f\left(y1\right)=\int_{y1}^{\infty }f_{X,Y}\left(x,y\right)\cdot dx=\int_{y1}^{y2}f_{X,Y}\left(x,y\right)\\ \;\cdot dx+\int_{y2}^{\infty }f_{X,Y}\left(x,y\right)\cdot dx>f\left(y2\right),\;\text{for}\;y1<y2. \end{array}$$

This implies that the probability density function of the reported lifespan is a decreasing function. In other words, there are more mature individuals with short, rather than long, reported lifespans. Thus, the fitted equation should be largely dependent on mortality risk at the start age of the senescent stage, rather than the mortality risk at a relatively old age, as there are smaller and smaller sample sizes for calculating mortality risk as organisms get older. We have demonstrated that the mortality risk at the start age of the senescent stage is overestimated (Equation 15). Thus, the likelihood of underestimating the increase in mortality risk against age based on the approach of fitting an equation is correspondingly high.

### 4.2 Biological significance

Understanding how mortality risk changes with age is crucial to the study of evolutionary biology, conservation science, and senescence (Baudisch, 2011). The change of mortality risk can be determined or reflected by the parameters in the aging models (Ricklefs & Scheuerlein, 2002; Ronget et al., 2020). Among the numerous aging models, Weibull and Gompertz models are widely employed, especially in mammals (Gavrilov & Gavrilova, 2015; Ronget et al., 2020) and birds (Pinder et al., 1978; Ricklefs & Scheuerlein, 2002). Parameter *c* in the Weibull model and parameter *λ* in the Gompertz model reflect the change in the age‐specific mortality risk. In theory, these parameters can be correctly estimated by observing lifespan from a sample of individuals.

For wild animals, lifespan is most often inferred from capture–recapture/recovery data (Catchpole et al., 1998). These data often include numerous records with missing information, which limits the inferences that can be drawn based on mortality risk patterns (Metcalf et al., 2009; Ricklefs & Scheuerlein, 2001). There are many methods for parameter estimation from two types of censoring data (Marshall & Olkin, 2007; Rinne, 2009): type–I censoring data (i.e., monitoring is suspended when a fixed time has been reached) and type–II censoring data (i.e., monitoring is suspended when a fixed number of failures has been reached). These censoring data are clearly linked to the study span. However, the situation is more complicated for biological research in which, in addition to the influence of the study span, missing information always results from the recovery probability (Baylis et al., 2014; Gimenez et al., 2008). The missing information in capture–recapture/recovery data cannot be treated as either type–I censoring data or type–II censoring data, as we do not know the lower limit (i.e., the fixed time in type–I censoring data) for the missing values (i.e., the lifespans of unrecaptured individuals), and whether the lifespans of unrecaptured individuals are larger than the lifespans of recaptured individuals (i.e., fixed number of failures in type–II censoring data).

Many analytical methods have been developed in the last two decades, allowing inferences about the age‐specific survival rates/mortality risk from capture–recapture/recovery data with missing information (Colchero et al., 2012). For example, Müller et al. (2004) and Baylis et al. (2018) developed analytical tools that can deal with imperfect detectability and unknown birth time, given that the accurate death time is known. Zajitschek et al. (2009) and Colchero and Clark (2012) developed analytical tools that account for records with missing birth and death times; however, the recovery probability should be sufficiently larger for estimating survival parameters in this method (Colchero & Clark, 2012). In the study conducted by Zajitschek et al. (2009), the probability of a marked individual being recaptured at least once was 99.97714% for males and 99.99994% for females in the total survey period (5984 person‐minutes).

For wild animals with high dispersal ability and/or small body size (e.g., bats, rodents, and most birds), the recovery rates are usually very low (Cleminson & Nebel, 2012). The only information for most of these species concerning senescence is the reported lifespan based on when individuals are last seen or caught (Moorad et al., 2012; Xia & Møller, 2021). We deduced the probability density function of the reported lifespan based on the framework of the Weibull and Gompertz models. The key point is that the probability density function of the reported lifespan is not in accordance with the Weibull or Gompertz distribution. If this difference is ignored, the magnitude of the increase in mortality is largely underestimated. This study provides an explanation for the negligible senescence of wild animals with low recovery probability. Furthermore, our work can provide an analytical tool for evaluating the aging rate if the real lifespan belongs to Weibull or Gompertz models, as the probability density function of the reported lifespan using the same parameters (*m*_0_, *b*, *c*, *θ*, *λ*) as in the Weibull and Gompertz models.

## 5 CONCLUSIONS

In conclusion, we show that the actuarial senescence (i.e., the increase in mortality against age) is largely underestimated based on the reported lifespan with low recovery probability, which can provide an explanation for the evidence of negligible or negative senescence in many wild animals. Humans attempt to obtain insights from other creatures to better understand our own biology and to determine how best to enhance and extend human health (Bernard et al., 2020; Jones et al., 2014). Our advice is that reports of negligible senescence in animals should be interpreted with caution and rigorously analyzed. Being able to escape from senescence is possibly a beautiful illusion.

## AUTHOR CONTRIBUTION

**Canwei Xia:** Conceptualization (equal); Data curation (equal); Formal analysis (lead); Funding acquisition (equal); Investigation (equal); Methodology (lead); Project administration (equal); Resources (supporting); Software (equal); Supervision (equal); Validation (equal); Visualization (equal); Writing – original draft (lead); Writing – review & editing (supporting). **Anders Pape Møller:** Conceptualization (equal); Data curation (equal); Formal analysis (supporting); Funding acquisition (equal); Investigation (equal); Methodology (supporting); Project administration (equal); Resources (equal); Software (equal); Supervision (equal); Validation (equal); Visualization (equal); Writing – original draft (supporting); Writing – review & editing (equal).

## CONFLICT OF INTERESTS

The authors declare that they have no competing interests.

## Data Availability

All data are available in the main text.
